# Stereotype Content at the Intersection of Gender and Sexual Orientation

**DOI:** 10.3389/fpsyg.2021.713839

**Published:** 2021-07-15

**Authors:** Amanda Klysing, Anna Lindqvist, Fredrik Björklund

**Affiliations:** ^1^Department of Psychology, Lund University, Lund, Sweden; ^2^Department of Psychology, Stockholm University, Stockholm, Sweden

**Keywords:** stereotype content model, gender, sexual orientation, stereotype content, intersectionality, agency, communion, implicit tests

## Abstract

According to the Stereotype Content Model (SCM), the content of stereotypes differs on two dimensions: communion and agency. Research shows that for stereotypes about the general gender categories of “women” and “men,” there is an ambivalent pattern of communion and agency, where high levels on one dimension are associated with low levels on the other. For sexual minority stereotypes, a gender inversion has been found, whereas homosexual women are seen as more similar to men in general than to women in general, whereas homosexual men are seen as more similar to women in general than to men in general. However, there is limited research on how stereotype content for general groups relate to stereotype content for subgroups with intersecting category memberships. This research addresses this gap by investigating stereotype content at the intersection of gender and sexual orientation, including stereotype content for general gender groups, heterosexual groups, homosexual groups, and bisexual groups. In Study 1, a community sample from Sweden (*N* = 824) rated perceived communion and agency for women and men in general, as well as hetero-, homo-, and bisexual women and men. In Study 2, a nationally representative Swedish sample (*N* = 424) performed the same rating task, and in addition completed Single-Category IATs (SC-IATs) for warmth and competence. Results from both studies show that the stereotype content for the general categories “women” and “men” overlap with the stereotype content for heterosexual same-gender targets. Homosexual and bisexual groups were rated as more similar to their non-congruent gender category than same gender heterosexual categories were, but stereotype content for sexual minority groups did not overlap with either general gender categories, thus showing only incomplete gender inversion of stereotype content. Implicit associations between “women” and “warmth” were significantly stronger than associations between “men” and “warmth.” There were no other significant relations between implicit associations to warmth/competence and gender or sexual orientation. Theoretical and methodological implications for future research into intersectional stereotype content are presented, including how the findings inform the co-dependent relationship between a binary gender structure and a heteronormative ideology.

## Introduction

Stereotypes are cognitive schemas that incorporate culturally shared representations of social groups and influence information processing related to social categorization (Dovidio et al., 2010; Yzerbyt, 2016). Stereotypes are thus both characteristics seen as common within a social group, for instance, “gay men are fashionable,” and something that influences social categorization, for instance, “because that man is fashionable he is probably gay” (Cox and Devine, 2015). Stereotype content has been found to generally vary along two dimensions of social content: agency and communion (Abele and Wojciszke, 2014; Fiske, 2019). Agency consists of social content relevant for goal achievement and task functioning, while communion consists of social content relevant to relationship maintenance and social functioning (Abele and Wojciszke, 2014). The Stereotype Content Model (SCM) provides a functional explanation for why stereotype content is organized into these two dimensions, suggesting that the degree of agency and communion is determined by societal status and perceived competitiveness of the group (Fiske et al., 2002; Caprariello et al., 2009; Durante et al., 2013; Kervyn et al., 2015). Communion and agency have previously been treated as distinct from the dimensions of warmth and competence, which are the dimensions that the SCM was built around, but later developments have established that the dimensions are parallel to each other. The dimensions that have been called warmth and communion are both made up of the facets warmth and morality, while the dimensions that have been called competence and agency are made up of the facets competence and assertiveness (Abele et al., 2016; Fiske, 2019). The terms communion and agency will be used throughout this article unless the intension is to address specific facets of these dimensions.

Within the SCM, special emphasis is placed on the combined evaluation of a social group’s communion and agency, with many groups displaying ambivalent stereotype content; for instance being seen as warm but not competent (Fiske et al., 2002). Expanding on the implications of stereotype content, the degree of communion and agency included in a group stereotype can then predict societal behavioral responses toward the group (Cuddy et al., 2007; Fiske et al., 2007; Caprariello et al., 2009; Kervyn et al., 2015; Bye and Herrebrøden, 2018). The SCM thus provides an explanatory framework connecting stereotype content to social structure, as well as to intergroup behaviors.

Stereotype content for a large number of groups in multiple cultures has been investigated using the SCM (see Fiske and Durante, 2016, for a review). However, there is limited research on how stereotype content for general groups relate to stereotype content for subgroups with intersecting category memberships, which also limits our knowledge about how social categories are jointly related to stereotype content. The stereotype content for sexual minority groups has been found to be partly inverted compared to the content of stereotypes about the general gender groups women and men, such that the stereotype content for lesbian women is more similar to that of men in general, while the stereotype content for gay men is more similar to that of women in general^1^ (see for instance, Blashill and Powlishta, 2009). However, the degree to which this represents an inversion of gender stereotypes, compared to an androgynous view of sexual minorities, is not clear. Furthermore, the degree to which this suggested inversion reflects proximity or distance to stereotype content for gender groups without specified sexual orientation has not been directly tested.

The current study, therefore, aims to provide an intersectional analysis of stereotype content for groups at the intersection of gender and sexual orientation. We expand upon previous research by including direct comparisons of stereotype content for general gender categories to that of intersecting subgroups, by including understudied sexual minority groups, and by testing stereotype content using both explicit and implicit measures.

### The Intersection of Gender and Sexual Orientation

A multitude of studies have identified that women are stereotyped as high in communion but low in agency, while men are stereotyped as low in communion and high in agency (for reviews, see, e.g., Heilman, 2001; Wood and Eagly, 2010; Ellemers, 2018). Several studies within the SCM framework have found support for the predicted ambivalence of general gender stereotypes in different cultures (e.g., Fiske et al., 2002; Cuddy et al., 2009, 2015; Asbrock, 2010; Bye et al., 2014). However, stereotype content related to gender varies over time (Eagly et al., 2020) and across nations (see for instance, Diekman et al., 2005; Cuddy et al., 2015; Sendén et al., 2019) with regards to both degree of communion and agency. In Sweden, for instance, recent data on gender stereotype content indicate that men are indeed stereotyped as lower in communion compared to women, but that women and men are rated as equally agentic (Sendén et al., 2019). Beyond culture and time, the content of gender stereotypes is further complicated by the connection between gender typicality and inferred sexual orientation.

Gender atypical behavior is used as a heuristic for classifying individuals as not heterosexual (see research on “gaydar,” e.g., Cox et al., 2016). In contrast, assertions of heterosexuality frequently make use of exaggerations of gender typical behavior, particularly for men (Bosson et al., 2012; Davis-Delano and Morgan, 2016). Research on beliefs regarding gender inversion of characteristics associated with sexual minorities have found that homosexual women and men are seen as more similar to other-gender heterosexual groups than to their respective same gender group (Kite and Deaux, 1987; Blashill and Powlishta, 2009), and that heterosexual groups are seen are more gender typical than homosexual or bisexual groups (Ghavami and Peplau, 2018). However, this “inversion” does not mean that sexual minority groups are viewed as the same as other-gender heterosexual groups, but rather as different from both their own gender group and the other included gender group (Blashill and Powlishta, 2009). In fact, homosexual women and men can be rated as equally masculine and feminine (Clarke and Arnold, 2017) with comparable degrees of similarities to both same and other gender groups (Ghavami and Peplau, 2018): suggesting an androgyny rather than gender inversion view of sexual minorities.

The typical SCM paradigm involves measuring stereotype content for a diverse sample of groups identified by participants as salient in a specific culture (see for instance, Fiske et al., 2002). Studies of stereotype content for sexual minority groups conducted in different cultures show different degrees of agency and communion included in cultural stereotypes regarding homosexual women and men, ranging from high on both dimensions (homosexual men in Norway; Bye et al., 2014) to low on both dimensions (homosexual men in Mexico; Durante et al., 2013). Findings from Australia (Durante et al., 2013), Germany (Eckes, 2002; Asbrock, 2010), Italy (Brambilla et al., 2011), and the United States (Fiske et al., 2002) show either medium levels of agency and communion included in the stereotypes about homosexual women and men, or partial gender inversion of stereotype content. Gay men have been more commonly mentioned in such group salience measures than lesbian women, potentially due to mechanisms of intersectional invisibility (Purdie-Vaughns and Eibach, 2008), and bisexual women and men are completely absent. Studies specifically dedicated to measuring stereotype content for homosexual and bisexual women and men (Vaughn et al., 2017), as well as heterosexual ditto (Mize and Manago, 2018), are few in number. Comparisons between homosexual and bisexual women and men indicate either that bisexual groups form a cluster relatively low on both communion and agency (Mize and Manago, 2018), or that ratings of communion follow a gendered (but inverted) pattern, while ratings of agency is lower for bisexual men than for remaining groups (Vaughn et al., 2017). Looking not across but within sexual minorities also reveals diametrically different stereotype content for subgroups of lesbian women and gay men (Clausell and Fiske, 2005; Brambilla et al., 2011).

Studies of social categories like gender can be fruitfully enriched by incorporating an intersectional framework, in which gender is not treated as an isolated factor of social categorization but rather a social positioning that gains meaning from a complex system of interrelations (Crenshaw, 1989; McCall, 2005; Cole, 2009). In this spirit, it has long been suggested that sexuality plays an intrinsic role in constituting gender as a meaningful category (see Connell, 1987; Wittig, 1992b; Butler, 2006 for a few influential examples). Gender as a binary structure relies on the existence of two groups with opposing qualities that complement each within heterosexuality, what Butler (2006) called the heterosexual matrix. An intersectional analysis that allows analysis of both how sexual minorities relate to general gender groups and how general gender groups depend on a specific sexual orientation for meaning therefore makes an investigation of the constitutive interplay of different dimensions of categorizations in relation to stereotype content possible. This has not been possible in the studies that have been reviewed here, so far, given that direct measures of general gender stereotypes have not been included. Instead, gender stereotypes have been assumed to conform to the pattern of women being stereotyped as low agency-high communion and men as high agency-low communion. To this end, the current research includes direct measures of general gender stereotypes in addition to measures of stereotype content for groups at the intersection of gender and sexual orientation. This study thus answers the call for a greater intersectional focus in psychological research on social groups (Cole, 2009; Nicolas et al., 2017) with a quantitative focus (Else-Quest and Hyde, 2015).

### Implicit Measures of Stereotype Content

Beyond using trait rating scales, stereotype content can also be measured implicitly, something that has been called for as a future research direction within SCM (Fiske, 2019). The most common implicit measure of associations between a target group and an attribute dimension is the Implicit Association Test (IAT; Greenwald et al., 1998). The IAT aims to measure the strength of the association between a target concept and an attribute dimension through reaction time latency (Fazio and Olson, 2003). The association between the target concept and attribute dimension is given relative to a complementary target concept, e.g., the association between White-Good relative to Black-Good rather than simply the association strength between White-Good (Greenwald et al., 1998). Similar to explicit stereotypes, women, compared to men, are more strongly implicitly associated to warmth (Ebert et al., 2014). However, unlike explicit associations, participants of either gender implicitly associate their own gender more strongly with competence (Ebert et al., 2014). Implicit gender stereotyping thus appear to follow the pattern of explicit gender stereotypes in that the stereotype for women contain more warmth/communion than that for men, while competence/agency is more variable (see for instance, Sendén et al., 2019; Eagly et al., 2020). To address the limitation that the IAT only measures relative associations, the Single-Category IAT (SC-IAT) was developed (Karpinski and Steinman, 2006). For stereotype content at the intersection of gender and sexual orientation, the SC-IAT could provide a valuable insight into implicit associations between complex social groups without obvious complementary categories and stereotype content dimensions. While implicit attitudes toward homosexual and bisexual women and men have begun to become an object of study (for examples, see for instance, Steffens and Wagner, 2004; Morrison et al., 2010; Breen and Karpinski, 2013), there are to our knowledge no studies on implicit stereotype content in terms of communion and agency for sexual minorities using the SC-IAT. This research represents a first exploration on if there is a relationship between explicit and implicit stereotypes at the intersection of gender and sexual orientation, thus providing valuable information on the degree of automaticity in associations between the stereotype dimensions of communion and agency and intersectional groups.

### Overview of the Current Study

The current research aimed to provide a description of stereotype content at the intersection of gender and sexual orientation, specifically for the genders women and men, and the sexual orientations heterosexuality, homosexuality, and bisexuality. To achieve this goal, two studies were conducted using an online community sample as well as a nationally representative sample. There are two main questions that this research informs: What is the gender stereotype content for groups at the intersection of gender and sexual orientation, and how does stereotype content for general gender categories relate to these subgroups?

## Materials and Methods

### Procedure

Participants in both studies were randomly assigned to respond to one of the following eight target groups: women, men, heterosexual women, heterosexual men, homosexual women, homosexual men, bisexual women, and bisexual men. Sample sizes for each condition varied from 89 to 121 in Study 1 and from 45 to 57 in Study 2, exact sample sizes for all conditions can be found in Supplementary Table S1.

The procedure for Study 1 consisted of participants rating their assigned group on communion and agency measures, followed by demographic items. For Study 2, participants first performed two SC-IATs (one for warmth and one for competence), and then followed the same procedure as in Study 1. Both studies were hosted on Qualtrics,^2^ and no personal data were collected. The present research was carried out in accordance with the Swedish national guidelines on ethical research (Swedish Research Council, 2017) and International Standards for Authors (Wager and Kleinert, 2011). Participants were informed that their participation was voluntary and anonymous and that no personal information would be collected.

### Participants

#### Sample 1

Participants were recruited during the spring of 2019 using advertising in various user groups on the social media site Facebook (e.g., in groups consisting of lay people with scientific interest, groups focused on specific hobbies, and groups intended for social interaction). A total of 824 participants completed the study with less than 20% missing values and make up the final sample. Participants used a free-text response to report gender and sexual orientation in order to avoid limiting the response options available (as recommended by Lindqvist et al., 2020), and the first author coded the responses into the categories found in Table 1. Other demographics measured consisted of age and occupation, and can be found in Table 1.

**Table 1:** Sample demographics for samples 1 and 2.

	Sample demographics	
	Sample 1 (*N* = 824)	Sample 2 (*N* = 424)
**Gender**^1^		
Women	62%_a_ (509)	54%_b_ (228)
Men	34%_a_ (280)	45%_b_ (189)
Non-binary individuals	2%_a_ (13)	0.5%_a_ (2)
Did not respond	2%_a_ (22)	1%_a_ (5)
**Age**^2^		
Min-Max	16–83	16–84
*M*	44.29_a_	48.71_b_
*SD*	13.54	17.58
Missing values	14	2
**Sexual orientation**		
Heterosexual	77%_a_ (638)	85%_b_ (362)
Homosexual	3%_a_ (25)	0.5%_b_ (2)
Bi- or pansexual	11%_a_ (87)	6%_b_ (24)
Asexual	1%_a_ (9)	0%_b_ (0)
Other	1%_a_ (7)	0.2%_a_ (1)
Did not respond	14%_a_ (58)	8%_a_ (35)
**Occupation**		
Employed	63%_a_ (514)	61%_a_ (256)
Student	12%_a_ (97)	9%_a_ (37)
Retired	10%_a_ (84)	24%_b_ (100)
Unemployed	2%_a_ (17)	2%_a_ (9)
Sick leave	6%_a_ (47)	1%_b_ (5)
Other	7%_a_ (53)	3%_b_ (14)
Did not respond	2%_a_ (12)	1%_a_ (3)

Different subscripts denote a significant inter-study difference between groups at *p* < 0.05.

1 Given the larger proportion of women than men in sample 1, a *χ*^2^ test for independence was performed to ensure the proportion of women to men was equal in all groups. No significant difference in gender proportion was found between the conditions, *χ*^2^(7) = 5.60, *p* = 0.60.

2 A Levene’s test showed that the variance in age was significantly different between the samples, *F*(1, 1,230) = 67.83, *p* < 0.001, so a Welch’s *t*-test was used rather than a Student’s *t*-test.

#### Sample 2

For Study 2, a sample representative of the Swedish population in terms of age, binary gender, and regional location was recruited in June 2019 through a web panel hosted by Enkätfabriken.^3^ A total of 424 participants completed the implicit measures, while 423 participants completed the explicit measures with less than 20% missing values. Complete demographics can be found in Table 1 along with information on demographics differences between samples 1 and 2, when applicable.

### Materials

#### Study 1

##### Agency/Communion Scale

Perceived agency and communion of each target group were measured using rating scales with traits representing each dimension. Participants were instructed to rate the target group according to how they believe society views the group (see e.g., Fiske et al., 2002). The scale ranged from 1 (*not at all*) to 5 (*a great deal*), and the traits were taken from both the warmth/competence literature (see for instance, Fiske et al., 2002) and the agency/communion literature (Abele et al., 2016) due to the great overlap between the constructs (Fiske, 2019). Translation into Swedish was performed by the first author and evaluated by the other authors. See Supplementary Table S2 for specific items used. Both the communion and the agency measure showed high internal reliability, *α* = 0.91 and *α* = 0.91, respectively.

#### Study 2

##### Single Category IAT

The SC-IAT is a modification of the IAT (Greenwald et al., 1998) that allows for testing of associations between a target category and an attribute category without the use of a comparison category (Karpinski and Steinman, 2006). Two SC-IATs were programmed into Qualtrics using a modified version of the code provided by Carpenter et al. (2019).

Participants completed two separate SC-IATs with the attribute categories warmth/cold and competence/incompetence, respectively. The SC-IATs were conducted in Swedish, and because there are no direct translations of the terms communion and agency, the terms warmth and competence were used as names for the attribute categories presented to participants. However, stimulus words for the dimensions included words related to the all facets of the larger concepts of communion and agency. The stimulus words used for the target groups followed recommendations from Steffens et al. (2008) and consisted of synonyms for the target groups, see Supplementary Table S3 for all stimuli words, and Supplementary Table S4 for a complete list of group name synonyms.

**Figure 1:**
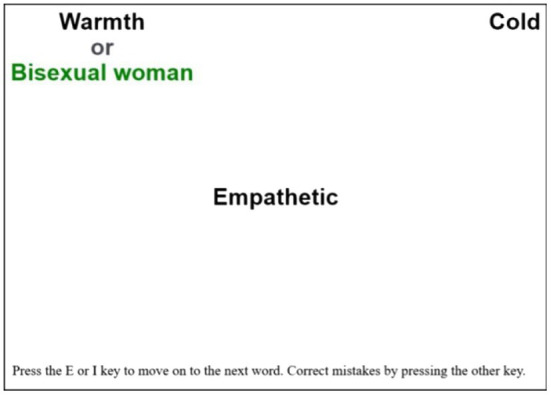
Trial from the Single-Category IAT (SC-IAT) comparing associations to bisexual women and warmth to associations between bisexual women and cold. In this permutation, bisexual women are paired with warmth, and in the comparison permutation bisexual women are paired with cold. Participants completed the SC-IAT in Swedish.

Participants completed the SC-IATs in a randomized order, with the order of the different potential placements of target groups and attribute categories randomized between left and right placement on the screen. Participants first completed a practice block consisting of 24 trials followed by a critical block of 60 trials. This was repeated a total of four times for each possible combination of target group and attribute category. Participants were instructed to sort the words appearing in the middle of the screen to one of the three categories available as fast as possible using the “e” and the “i” key. If an incorrect classification was performed, a red X appeared on the screen for 300 ms and the participant had to correct their classification before the test continued. See Figure 1 for an example of one trial.

##### Agency/Communion Scale

After performing the SC-IATs, participants rated their target group’s perceived agency and communion using identical rating scales as in Study 1, which displayed high internal reliability (*α* = 0.95 and 0.91, respectively).

## Results

The potential effect of participant gender was tested but was not significant in any analyses and is therefore excluded from reporting. Data analysis was conducted using R version 3.6.2 (R Core Team, 2019). ANOVAs were conducted using Type III sum of squares through the *car* package (Fox and Weisberg, 2019), bivariate confidence intervals were calculate using the package *jocre* (Pallmann, 2017), and split-half reliabilities for the SC-IATs were calculated using the package *multicon* (Sherman, 2015). All pairwise comparisons were performed using a Tukey HSD correction for multiple comparisons unless otherwise stated.

### Study 1

There was no significant correlation between ratings of agency and communion, *r* = − 0.05, *p* = 0.16, and descriptive data by group for both outcome variables can be found in Table 2. Two ANOVAs were performed to analyze differences in communion and agency based on group gender and group sexual orientation. There was a significant interaction effect between group gender (women, men) and group sexual orientation (none listed, heterosexual, homosexual, and bisexual) for both communion, *F*(3,816) = 83.93, *p* < 0.001, *η_p_*^2^ = 0.24, and agency, *F*(3,816) = 108.62, *p* < 0.001, *η_p_*^2^ = 0.29.

**Table 2:** Descriptive data for communion and agency by target group for Study 1.

	Women (*n* = 94)	Men (*n* = 109)	Heterosexual women (*n* = 89)	Heterosexual men (*n* = 89)	Homosexual women (*n* = 100)	Homosexual men (*n* = 111)	Bisexual women (*n* = 104)	Bisexual men (*n* = 96)
**Communion**								
*M*	3.74_a_	2.74_b_	3.71_a_	2.79_bc_	3.04_c_	3.43_d_	3.00_c_	3.37_d_
*SD*	0.56	0.58	0.56	0.67	0.64	0.55	0.71	0.53
**Agency**								
M	2.73_a_	3.93_b_	2.92_ad_	3.71_bc_	3.58_c_	2.83_ad_	3.28_e_	3.03_de_
SD	0.60	0.70	0.68	0.75	0.58	0.51	0.69	0.48

All variables have a range from 1 to 5. Different subscripts denote a significant difference between groups at *p* < 0.05 for each dimension. n denotes number of participants that rated each target group.

Group differences based on pairwise comparisons for the interaction effect on communion are shown in Table 2. There was gender stereotype congruence for the general gender categories as well as the heterosexual categories: i.e., “women” were rated as more communal than “men” (*d* = 1.76), and heterosexual women were rated as more communal than heterosexual men (*d* = 1.48). “Women” and heterosexual women did not significantly differ from each other (*d* = 0.06) and were both rated as more communal than all other categories (*ds* < 1.76, > 0.51). For non-heterosexual categories, the pattern of gender stereotypes was reversed: homosexual men and bisexual men were rated as more communal than homosexual women and bisexual women, respectively (*ds* = 0.65 and 0.58).

Group differences based on pairwise comparisons for the interaction effect on agency are shown in Table 2. There was gender stereotype congruence for the general gender categories as well as the heterosexual categories: i.e., “men” were rated as more agentic than “women” were (*d* = 1.83), and heterosexual men were rated as more agentic than heterosexual women were (*d* = 1.11). “Men” were rated as more agentic than all other categories (*ds* < 1.83, > 0.54), except heterosexual men (*d* = 0.29). Heterosexual men were rated as more agentic than all remaining categories except homosexual women (*d* = 0.20). For non-heterosexual categories, the pattern of gender stereotypes was partially reversed. Homosexual women were rated as more agentic than homosexual men (*d* = 1.37), but bisexual women were not rated as significantly more agentic than bisexual men (*d* = 0.41).

Paired *t*-tests were performed to determine intragroup differences in communion and agency, analysis details of which can be found in Supplementary Table S4. The following groups were rated as more communal than agentic: “women,” heterosexual women, homosexual men, and bisexual men. “Men,” heterosexual men, homosexual women, and bisexual women were rated as more agentic than communal.

**Figure 2:**
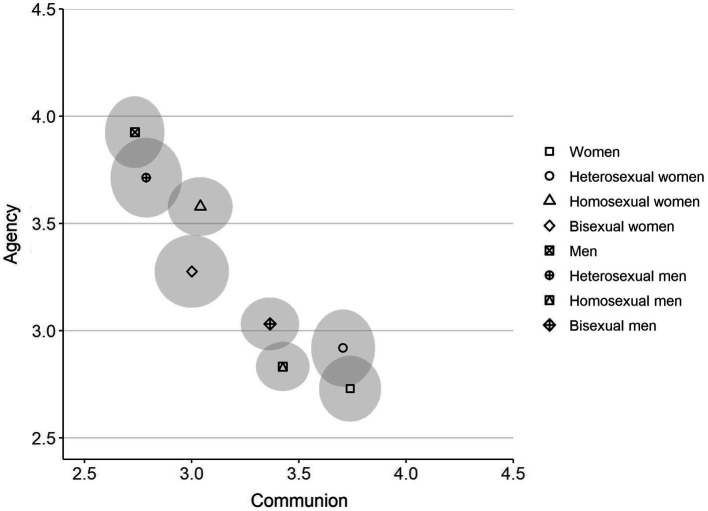
Mean ratings of agency and communion by target group for Study 1. Ellipses represent the 95% bivariate confidence region around the multivariate mean for each group. The axes have been truncated for clarity; full range of both scales is 1–5.

Finally, the communion and agency ratings for each group were mapped into a two-dimensional space in order to investigate both how the groups compare to each other and how they place in the SCM framework of ambivalent stereotypes. To give an estimate for the relation between groups in the bivariate space, a standard bivariate confidence region for each group’s mean value of agency and communion was calculated (see Chew, 1966; Pallmann and Jaki, 2017 for specific method). As can be seen in Figure 2, the groups ranged from high agency-low communion to low agency-high communion with no groups placing in the high-high or low-low quadrant. There was an overlap between the confidence regions for the following pairs: “men” and heterosexual men, “women” and heterosexual women, heterosexual men and homosexual women, and homosexual men and bisexual men. The two bisexual categories placed closest to the middle of the map, and as such displayed no notable gender inversion.

### Discussion

Study 1 showed that gender stereotypes in this sample were ambivalent and complementary, in terms of both communion and agency: “women” were stereotyped as high in communion but lower in agency and “men” were stereotyped as high in agency but lower in communion. This finding is in line with previous studies within an SCM paradigm (e.g., Fiske et al., 2002; Cuddy et al., 2009, 2015; Asbrock, 2010; Bye et al., 2014), but stands in contrast to previous data from Sweden, which showed no difference in the degree of agency in stereotypes about women and men (Sendén et al., 2019). One reason for this discrepancy could be that the studies have used different participant instructions, asking for cultural vs. personal estimations, respectively. Compared to cultural stereotypes, personal stereotypes have been shown to be higher in the depreciated dimension for groups with ambivalent stereotype content (e.g., communion for men), but not differ in terms of the higher rated dimension (e.g., agency for men; Kotzur et al., 2020). It is unclear, however, if this difference is due to social desirability concerns or a genuine discrepancy between cultural and personal stereotypes. Compared to Sendén et al. (2019) who asked about personal views on which traits women or men were likely to display, the current study only partly follows this pattern: personal stereotypes about “women” contain higher degrees of agency than cultural stereotypes, but personal stereotypes about “men” do not seem to be higher in communion than cultural stereotypes.

When sexual orientation is specified, stereotype content for sexual minority groups differs from the stereotype content for their respective general gender group, but this is not the case for heterosexual groups. It thus appears that general gender stereotypes only apply to heterosexual women and men. A similar pattern has been shown for the intersection of gender and ethnicity, such that stereotypes about women and men only matched those about White women and men, while stereotypes about Black people only matched those of Black men (Ghavami and Peplau, 2013).

The way that stereotype content for sexual minority groups differs from general gender stereotypes shows a partial gender inversion. As stated, all sexual minority groups show a difference to both the gender congruent and gender incongruent general gender group, so it is not the case that for instance, the stereotype for homosexual men is the same as that for women in general. However, all sexual minority groups showed a greater similarity to the gender incongruent general gender group than to the gender congruent one. Homosexual and bisexual men showed a similar degree of agency as heterosexual women, and homosexual and bisexual women showed a similar degree of communion as heterosexual men. Homosexual and bisexual men differed from heterosexual women in terms of communion, and while bisexual women differed from heterosexual men in terms of agency, homosexual women did not. As an overall pattern, this data indicate that sexual minorities receive lower ratings for the gender congruent dimension (communion for women and agency for men), but the corresponding increase in the gender incongruent dimension is not comparable in size to that of heterosexual groups. That is, bisexual women are as low in communion as heterosexual men, but not as high in agency. The exception to this pattern is homosexual women who were indeed seen as similar to heterosexual men in terms of both communion and agency, but still differed from men in general. These results thus fall somewhere in between a gender inversion (e.g., Kite and Deaux, 1987; Blashill and Powlishta, 2009) and an androgyny interpretation of stereotypes about sexual minorities (Clarke and Arnold, 2017; Ghavami and Peplau, 2018).

Looking within sexual minority groups, gender seems to be the organizing factor: homosexual men and bisexual men cluster together, while homosexual women and bisexual women are similar in communion but somewhat different in agency. This differs somewhat from previous studies that have found that stereotype content for homosexual women and men are more similar to gender incongruent heterosexual groups, while stereotype content for bisexual women and men cluster together to a greater degree (Vaughn et al., 2017; Mize and Manago, 2018). A key difference between the current study and previous studies that have included bisexual groups is that ratings in the current study are conducted completely between-groups, whereas previous studies have been either completely within-participant designs (Vaughn et al., 2017) or had a between-within design (Mize and Manago, 2018). Collecting completely independent stereotype content ratings thus appear to indicate that gender identity and sexual orientation dynamically influence stereotype content, such that gender identity organizes stereotype content within sexual minority groups, while sexual orientation organizes stereotype content within gender groups. This pattern is consistent with an interpretation of gender and sexual orientation being co-constitutive in terms of associated traits rather than two independent categories.

### Study 2

There was a significant, positive correlation between ratings of agency and communion, *r* = 0.44, *p* < 0.001, and descriptive data by group for both outcome variables can be found in Table 3. Two ANOVAs were performed to analyze group differences in communion and agency based on group gender and group sexual orientation. There was a significant interaction effect between group gender (women, men) and group sexual orientation (none listed, heterosexual, homosexual, and bisexual) for both communion, *F*(3,415) = 14.27, *p* < 0.001, *η_p_*^2^ = 0.09, and agency, *F*(3,415) = 7.95, *p* < 0.001, *η_p_*^2^ = 0.06.

**Table 3:** Descriptive data for communion and agency by target group for Study 2.

	Women (*n* = 56)	Men (*n* = 53)	Heterosexual women (*n* = 54)	Heterosexual men (*n* = 53)	Homosexual women (*n* = 54)	Homosexual men (*n* = 45)	Bisexual women (*n* = 57)	Bisexual men (*n* = 51)
**Communion**								
*M*	3.96_a_	3.22_b_	3.79_ac_	3.22_b_	3.45_bc_	3.44_bc_	3.22_b_	3.35_b_
*SD*	0.56	0.69	0.69	0.60	0.59	0.54	0.63	0.53
**Agency**								
*M*	3.36_b_	3.79_a_	3.41_b_	3.59_ab_	3.50_abc_	3.18b_cd_	3.20b_cd_	3.15_bd_
*SD*	0.63	0.50	0.65	0.69	0.59	0.48	0.54	0.48

All variables have a range from 1 to 5. Different subscripts denote a significant difference between groups at *p* < 0.05 for each dimension. n denotes number of participants rating each target group.

Group differences based on pairwise comparisons for the interaction effect on communion are shown in Table 3. There was gender stereotype congruence for the general gender categories as well as the heterosexual categories: i.e., “women” were rated as more communal than “men” (*d* = 1.20), and heterosexual women were rated as more communal than heterosexual men (*d* = 0.87). “Women” were rated as more communal than all other categories (*ds* < 1.28, > 0.88), except heterosexual women (*d* = 0.27). Heterosexual women were rated as significantly more communal than bisexual women and men (*d* = 0.86 and 0.70).

Group differences based on pairwise comparisons for the interaction effect on agency are shown in Table 3. There was gender stereotype congruence for the general gender categories: i.e., “men” were rated as more agentic than “women” (*d* = 0.77). “Men” were also rated as more agentic than heterosexual women, homosexual men, bisexual women, and bisexual men (*ds* = 0.66, 1.26, 1.15, and 1.31). Heterosexual men significantly differed from homosexual men, bisexual women, and bisexual men (*ds* = 0.68, 0.64, and 0.74).

Paired *t*-tests were performed to determine intragroup differences in communion and agency, analysis details of which can be found in Supplementary Table S4. The following groups were rated as more communal than agentic: “women,” heterosexual women, homosexual men, and bisexual men. “Men” and heterosexual men were rated as more agentic than communal. There was no significant difference in agency and communion for homosexual or bisexual women.

**Figure 3:**
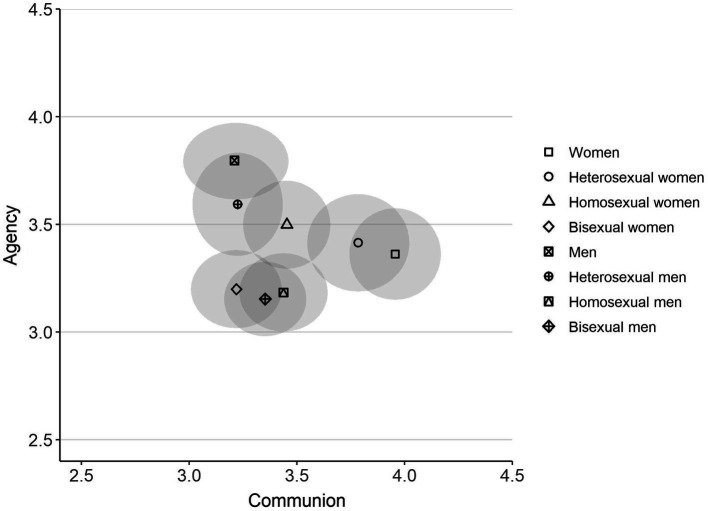
Mean ratings of agency and communion by target group for Study 2. Ellipses represent the 95% bivariate confidence region around the multivariate mean for each group. The axes have been truncated for clarity; full range of both scales is 1–5.

Finally, the communion and agency ratings for each group were mapped into a two-dimensional space. As can be seen in Figure 3, the overlap between groups is greater in Study 2 than in Study 1 and in general the groups place closer to the middle of the map. However, the category of “women” still only overlapped with heterosexual women, and “men” only overlapped with heterosexual men and homosexual women. Remaining groups all showed some two-dimensional overlap with each other.

To test the difference in ratings of communion and agency between samples, two (group gender: women, men) × 4 (sexual orientation: none listed, heterosexual, homosexual, and bisexual) × 2 (sample: Study 1, Study 2) ANOVAs were conducted. There were significant three way interaction effects for both communion, *F*(3, 1,231) = 6.44, *p* < 0.001, *η_p_*^2^ = 0.02, and agency, *F*(3, 1,231) = 16.20, *p* < 0.001, *η_p_*^2^ = 0.04. Pairwise comparisons between samples showed that ratings of communion was higher in Study 2 compared to Study 1 for “men,” heterosexual men, and homosexual women (*p*s < 0.01). For agency, ratings were higher in Study 2 compared to Study 1 for “women” and heterosexual women (*p*s < 0.001).

Data cleaning of reactions times for the SC-IAT was performed following recommendations from Greenwald et al. (2003) and Karpinski and Steinman (2006). Participants with more than 10% of responses faster than 300 ms or over 20% error rate were excluded, as were trials slower than 10,000 ms. Participants with a mean reaction time between two and three SDs above the target group mean were excluded as outliers. Details about number of participants and trials excluded per SC-IAT can be found in Supplementary Table S6.

Reliabilities for the SC-IATs were calculated for the response times for warmth-cold and competence-incompetence separately for each target group. Split-half reliability was calculated from 1,000 split-half correlations and averaged between groups. The internal reliability for both the warmth-cold SC-IAT and the competence-incompetence SC-IAT showed reliability correlations comparable to previous research using SC-IAT measures, *r* = 0.75 (*SD* = 0.15) and *r* = 0.82 (*SD* = 0.14), respectively (see Greenwald and Lai, 2020 for comparable reliability correlations).

Single-Category IAT results were calculated in the form of *D*-scores based on the scoring algorithm developed in Greenwald et al. (2003) with the specific SC-IAT method described in Karpinski and Steinman (2006). For the Warmth-Cold SC-IAT, participant average response times for the block Group + Warmth was subtracted from averages for Group + Cold. This value was then divided by the pooled SD for all response times for both blocks to create a warmth-cold *D*-score with positive values indicating faster associations to warmth than to cold. The same procedure was followed for the Competence-Incompetence SC-IAT. A stereotype content *D*-score was then created by subtracting the Warmth-Cold *D*-score from the Competence-Incompetence *D*-score. Positive values of the stereotype content *D*-score indicate faster associations to competence (relative to incompetence) than to warmth (relative to cold). Descriptive data for *D*-scores divided by target group can be found in Table 3 and descriptive data for reaction times can be found in the Supplementary Table S7. As can be seen in Table 4 from the 95% CIs for the group mean *D*-scores, all groups except “men” and homosexual men showed a stronger association to warmth compared to cold, while all groups except homosexual men and bisexual men showed a stronger association to competence compared to incompetence. No groups showed a stronger association to the negative dimensions (cold and incompetence) compared to the positive dimensions (warmth and competence). The only group whose CI for the mean stereotype *D*-score did not include zero was the group “men”; indicating that they elicited comparatively stronger associations to competence than to warmth.

**Table 4:** Means, SDs, and 95% CIs for Warmth-Cold, Competence-Incompetence, and stereotype content *D*-scores by target group.

	Warmth-Cold		Competence-Incompetence		Stereotype content	
	*M* (*SD*)	95% *CI*	*M*	95% *CI*	*M*	95% *CI*
Women	0.17 (0.26)	0.10, 0.24	0.15 (0.23)	0.09, 0.22	−0.02 (0.29)	−0.11, 0.07
Men	0.01 (0.19)	−0.04, 0.07	0.12 (0.29)	0.03, 0.20	0.11 (0.33)	0.01, 0.21
Heterosexual women	0.14 (0.26)	0.06, 0.22	0.19 (0.22)	0.13, 0.26	0.06 (0.26)	−0.02, 0.14
Heterosexual men	0.14 (0.23)	0.07, 0.21	0.14 (0.32)	0.05, 0.24	−0.002 (0.38)	−0.12, 0.12
Homosexual women	0.09 (0.19)	0.03, 0.14	0.12 (0.27)	0.05, 0.20	0.05 (0.28)	−0.04, 0.13
Homosexual men	0.05 (0.27)	−0.04, 0.14	0.03 (0.28)	−0.05, 0.12	−0.04 (0.29)	−0.13, 0.06
Bisexual women	0.09 (0.20)	0.03, 0.14	0.14 (0.25)	0.07, 0.22	0.06 (0.29)	−0.03, 0.15
Bisexual men	0.10 (0.26)	0.02, 0.18	−0.003 (0.24)	−0.08, 0.07	−0.10 (0.34)	−0.21, 0.004

95% CIs for the means are calculated from the *t*-distribution.

There was a significant, positive correlation between *D*-scores for Warmth-Cold and Competence-Incompetence (*r* = 0.20, *p* < 0.001), but no significant correlations between explicit ratings of communion or agency and either *D*-score (*r* = −0.07 and −0.02 for Warmth-Cold and *r* = −0.01 and 0.03 for Competence-Incompetence, *p*s > 0.57).

Three 2 (group gender: woman, man) × 4 (sexual orientation: none listed, heterosexual, homosexual, bisexual) ANOVAs were conducted to analyze differences in *D*-scores for Warmth-Cold, Competence-Incompetence and Stereotype Content based on target group. The effects of participant gender and version order on *D*-scores were tested to ensure that no unintended influence had occurred. Neither version order nor participant gender had a significant influence on any of the *D*-scores and will therefore not be reported.

For Warmth-Cold *D*-scores, there was a significant effect of group gender, *F*(1,359) = 10.46, *p* = 0.001, *η_p_*^2^ = 0.03. There was no significant main effect of group sexual orientation, *F*(3,359) = 1.15, *p* = 0.21. There was however an interaction effect of group gender and sexual orientation that bordered significance, *F*(3,359) = 2.57, *p* = 0.05, *η_p_*^2^ = 0.02. The nearly significant interaction effect was a result of a significant difference in Warmth-Cold *D*-scores between the groups “women” and “men” (*M*_diff_ = 15, *p* = 0.03), and only between these two groups. The difference indicates that the group “women” were associated significantly more strongly with warmth (relative to cold) than the group “men”.

For Competence-Incompetence *D*-scores, there was no significant effect of neither group gender, *F*(1,371) = 0.49, *p* = 0.48, sexual orientation, *F*(3,371) = 0.51, *p* = 0.68, nor a significant interaction between the two, *F*(3,371) = 0.88, *p* = 0.45.

For the combined Stereotype Content *D*-scores, there was a significant effect of group gender, *F*(1,343) = 4.19, *p* = 0.04, *η_p_*^2^ = 0.01. There was no significant main effect of group sexual orientation, *F*(3,343) = 0.78, *p* = 0.51. There was a significant interaction effect between group gender and sexual orientation, *F*(3,343) = 3.72, *p* = 0.02, *η_p_*^2^ = 0.03. The interaction effect of gender and sexual orientation was a result of a group difference between “men” and bisexual men (*M*_diff_ = −0.21, *p* = 0.03), and only these groups. This difference indicates that bisexual men were seen as having a greater difference in warmth (relative to cold) and competence (relative to incompetence) associations than men in general. As can be seen in Table 3, this was driven by “men” having a stronger association with competence while bisexual men had a stronger association with warmth.

### Discussion

Study 2 showed concurrent results with Study 1 regarding the presence of ambivalent explicit stereotype content for “women” and “men,” as well as regarding the overlap between stereotype content for general gender groups and gender congruent heterosexual groups. However, the degree to which stereotype content for sexual minority groups differ from heterosexual groups was smaller in Study 2 compared to Study 1. There was no significant difference for neither communion nor agency between sexual minority groups nor between sexual minority groups and heterosexual men. Heterosexual women received higher communion ratings than sexual minorities and heterosexual men, but did not differ from these groups in terms of agency.

Stereotype content for sexual minority groups differed less from stereotype content for heterosexual groups in Study 2 than in Study 1. Homosexual men, bisexual men, and bisexual women received lower ratings of agency than heterosexual men did, but did not differ from heterosexual women. Bisexual women received lower ratings of communion than heterosexual women did, whereas homosexual women did not differ significantly from either heterosexual group in terms of either dimension. The overall pattern of the content of stereotypes about sexual minority groups thus showed very little gender inversion compared to the findings of Study 1. However, this is not a result of differences between studies in measured stereotype content for sexual minority groups but rather for general gender groups and heterosexual groups. Sexual minority groups in fact showed only one difference between the two samples: homosexual women were viewed as higher in agency in Study 2 compared to Study 1. For the remaining three groups stereotype content appeared rather constant. Instead, general gender groups and heterosexual groups received higher ratings in Study 2 compared to Study 1 on their respective depreciated dimensions. With ratings of communion and agency being more similar for the general gender groups and the heterosexual groups, the overlap with the static sexual minority groups naturally increased. However, even with these increases in agency and communion for “women” and “men,” respectively, the stereotype content for the general gender groups was still only concurrent with that of same gender heterosexual groups.

Group differences in implicit associations to stereotype dimensions were scarce, and did not follow the same patterns as explicit stereotype content. “Women” were associated more strongly with warmth than “men,” and bisexual men were associated more strongly with warmth than competence compared to “men.” However, implicit associations between target group and warmth only differed for the general gender groups, and was not present for any of the sexual orientation subgroups. Furthermore, there was no significant correlation between implicit and explicit measures of stereotype content. That “women” were more strongly associated with warmth (relative to both cold and competence) than “men” supports previous findings of an implicit women-warmth association (Ebert et al., 2014). However, the current study could not find the own-gender bias for implicit associations to competence (i.e., that women associate women more strongly with competence and men associate men more strongly with competence), which has previously been reported (Ebert et al., 2014).

Unlike previous research using the IAT to measure implicit associations to warmth and competence, the current study could not find any indication of ambivalent associations where groups are either associated with warmth or competence (cf. Carlsson and Björklund, 2010; Lindqvist et al., 2017), or the presence of implicit associations with an overall negative valence (cf. Rohmer and Louvet, 2012, 2018). Instead, participants showed overall stronger associations between the included groups and the positive rather than the negative dimensions, with the exception of sexual minority men who did not show a differential association between dimensions. One possible reason for this overall stronger association with positive dimensions could be the unbalanced valence of the paired dimensions in each SC-IAT. That is, it is possible that groups are more strongly associated with either warmth or competence without necessarily being associated with their respective negative counterparts. Previous research have shown that dimensions with unbalanced valence can be used to measure more negative implicit associations for the groups “the poor” and “the disabled” (Rohmer and Louvet, 2012, 2018; Lindqvist et al., 2017), indicating that the use of stimuli with negative valence should not in itself present an issue if there are genuinely negative implicit associations present. However, it may be the case that attitudes toward groups at the intersection of gender and sexual orientation influence implicit association to a greater degree than they do explicit measures of cultural stereotype content. Such a potential effect of attitude has been found for implicit associations between warmth/competence and housewives/businesswomen when measured with the lexical decision test, a different implicit measure of associations than the IAT. Women have been found to have stronger associations between businesswomen and positive terms in general regardless of their connection to warmth or competence, while men have stronger associations between housewives and positive terms (Wade and Brewer, 2006). That is, participants’ personal positive attitudes to the target groups in the current study could have resulted in valence and not attribute dimension guiding their implicit associations.

## General Discussion

The current findings showed that the stereotype content for the general gender categories “women” and “men” was similar to that of the specific categories heterosexual women and men, while being significantly different from that of homo- or bisexual women and men. As regards the gender inversion theory of sexual orientation (Kite and Deaux, 1987), the content of stereotypes about homosexual and bisexual categories were less gender stereotypical than the content of stereotypes about the general gender categories and the heterosexual categories. However, there was still a significant difference between stereotype content for the general gender categories and the corresponding gender-incongruent homosexual and bisexual category for both communion and agency. This indicates that the content of stereotypes about sexual minorities is not so much gender inversed as generally more androgynous than that of heterosexual counterparts.

Explicit stereotype content for the sexual minority groups was similar for both the community sample and the nationally representative sample, but stereotype content for general gender groups and heterosexual groups differed. Implicit associations to warmth and competence showed a stronger association between “women” and warmth, compared to “men” and warmth. However, there were no group differences found in terms of implicit associations to competence.

Early studies within an SCM framework that included sexual minority groups found that the stereotype content for the groups homosexual men and women was neutral rather than gender inversed (Fiske et al., 2002; Asbrock, 2010; Brambilla et al., 2011). This lack of ambivalent stereotype content connected to homosexual groups was suggested to be a result of contrasting stereotype content for salient subgroups of homosexual women and men leading to stereotype content ratings of medium agency and communion (Clausell and Fiske, 2005; Brambilla et al., 2011). Degree of gender conformity or gender non-conformity seems to be an organizing feature in perceptions of subgroups of lesbian women and gay men (Geiger et al., 2006; McCutcheon and Morrison, 2021), which could be one reason as why to subgroups of sexual minorities can be associated with contrasting stereotype content. However, the current studies find no indication that contrasting stereotype content for subgroups influenced the overall stereotype content of sexual minority groups, and instead falls in line with previous findings regarding the partial gender inversion of the content of stereotypes about homosexual individuals. Furthermore, Mize and Manago (2018) found that the gender inversion of stereotype content associated with sexual minorities might be more pronounced for sexual minority men than for sexual minority women. This was indicated by the presence of an overlap between participant ratings of both communion and agency for heterosexual women and homosexual men, whereas only ratings of communion overlapped for heterosexual men and homosexual women. However, the current research tells a different story with homosexual men being rated significantly lower regarding communion that heterosexual women in Study 1 but not in Study 2, while ratings of homosexual women did not differ from ratings of heterosexual men in terms of communion of agency in either study. As evidenced by these conflicting findings, sexual orientation and gender has a dynamic rather than simple relationship, and future research is needed to investigate how notions of gender inversion influences societal treatment of both sexual minority women and men.

The content of stereotypes for bisexual groups was more similar to the stereotype content for their respective gender congruent homosexual counterparts than to heterosexual or general groups, indicating that there may be a higher degree of gender atypicality in stereotype content for non-heterosexual groups in general and not exclusively for homosexual groups. This view of bisexual groups as more gender typical than homosexual groups, but less gender typical than heterosexual groups, coincides with findings from the United States regarding perceptions of bisexual women and men (Vaughn et al., 2017; Ghavami and Peplau, 2018; Mize and Manago, 2018). Note also that neither the stereotype content for bisexual women nor for bisexual men was low in communion or agency, meaning that stereotypes about these groups do not fall in the low-low corner of the SCM map that is associated with harmful behavioral responses from society (Cuddy et al., 2008). In fact, the lowest stereotype content dimension for bisexual women and men, respectively (communion and agency), was not significantly different from that of gender incongruent heterosexual groups. Recent national data from the United States show a similar pattern of a shift from very negative attitudes toward bisexuality (Herek, 2002) to generally neutral attitudes (Dodge et al., 2016). Findings from Germany show that attitudes in the general public were neutral with regards to bisexuality, even though subgroups of the population did hold negative attitudes (Steffens and Wagner, 2004). One potential reason for these differences in attitudes toward bisexual individuals could be that participants have different views of what bisexuality entails. There is a common prejudice in relation to bisexuality that bisexuality is not a valid sexual orientation unto itself, but rather an expression of confusion from people who are “actually” homosexual or heterosexual (Israel and Mohr, 2004; Hubbard and de Visser, 2015; Burke and LaFrance, 2016b; Morgenroth et al., 2021). Whether or not a bisexual person is seen as latently homosexual or heterosexual depends on their gender, such that bisexual women are seen as latently heterosexual and bisexual men are seen as latently homosexual (Flanders and Hatfield, 2013; Mize and Manago, 2018; Morgenroth et al., 2021). Because the stereotype content reported in the current study for bisexual groups was closer to that of gender congruent homosexual groups for both bisexual women and men, there is some indication that bisexual men were viewed as latently homosexual, but no indication that bisexual women were viewed are latently heterosexual.

In general, the current study did not find a clear relationship between explicit and implicit measures of stereotype content, except for the finding of stronger implicit association between “women” and warmth compared to “men.” This further supports the women-warmth association previously found (Ebert et al., 2014). However, this was not due to any specific subgroup of women being more strongly associated with warmth, but rather shows that implicit warmth associations to the general group “women” may not be present when sexual orientation is specified. A lack of general warmth associations with subgroups of women has also previously been found in relation to the groups “homemakers” and “businesswomen,” where participant gender instead made either group associated more strongly with overall positive attributes (Wade and Brewer, 2006). Participant gender did not have such an effect in the current study, nor did it influence implicit associations to competence in the pattern of own-gender bias that has been previously identified (Ebert et al., 2014). Furthermore, unlike previous studies using the IAT to study ambivalent stereotype content (Carlsson and Björklund, 2010; Lindqvist et al., 2017), our data shows no significant difference in implicit associations with warmth compared to competence. One potential reason for the discrepancy in studies of ambivalent implicit associations could be the structure of the group under investigation. In the current study, the target groups were subgroups of the larger groups of women/men and heterosexual/homosexual/bisexual individuals (in addition to the two general gender groups “women”/“men”). These groups are all made up of a combination of two social categories and were named as such in the materials presented to participants (e.g., homosexual woman). This differs from the group structure previously studied in regards to ambivalent implicit associations that have included groups with only one named social identity: lawyers/preschool teachers (Carlsson and Björklund, 2010), women/men (Ebert et al., 2014), and homemakers/businesswomen (Wade and Brewer, 2006). Using noun forms of groups names in this way has been found to heighten the salience of group membership, compared to using adjective forms (Graf et al., 2012). It is, therefore, possible that implicit associations to sexual minority groups named using nouns (e.g., lesbian) as opposed to adjectives (e.g., homosexual woman) would show a pattern of ambivalent implicit associations more similar to those found using explicit measures.

### Limitations

The current study included a sample recruited through social media, a vector of recruitment that is still relatively unstudied. There has been some concern raised that social media recruitment is particularly vulnerable to self-selection effects (Thornton et al., 2016), which could be one reason why there was greater polarization of evaluations in the social media sample compared to the online panel sample. It is worth noting that the use of social media recruitment provides access to more diverse participants than a student sample does (Hays et al., 2015), which was also the case in the current study in relation to sexual orientation of participants. However, even with a more diverse sample, the current study did not include a sufficient number of homosexual and bisexual participants to conduct analyses of potential intersectional ingroup effects. Future studies on stereotype content should investigate further whether or not stereotype content, and particularly implicit stereotype content, differs between members of different intersections of gender and sexual orientation. This may be particularly crucial for implicit stereotype content, since previous research has found implicit ingroup preferences related to sexual orientation (Steffens, 2008; Anselmi et al., 2013; Steffens et al., 2013).

To capitalize on theoretical developments identifying the person perception dimensions warmth and competence as parallels to communion and agency (Fiske, 2019), the trait rating scales used in the current study was substantially more extensive than those used in the previous SCM literature. Traits included covered all relevant facets of agency and communion (Abele et al., 2016), and showed high internal reliability. However, the scales do need to undergo dedicated psychometric testing to further determine their degree of construct validity.

A methodological limitation of using the SC-IAT online is a lack of control over the study environment. Since the SC-IAT requires a high degree of concentration from participants, the potential for environmental distractions to influence performance is considerable, and may be one contributing factor to the discrepancy between explicit and implicit stereotype content. A methodological contribution of the current approach; however, is the effective participant recruitment that potentially lessened the influence of self-selection biases, in that it became less strenuous for participants to complete the test compared to in-person testing.

### Suggestions for Future Research

In the current study, bisexual women and men showed neither high nor low degrees of either stereotype dimension. Previous research into stereotypes about bisexual people have found a lack of knowledge among heterosexual individuals about traits associated with bisexual women and men (Zivony and Lobel, 2014), while homosexual and bisexual individuals report stereotypes closer to those about homosexual groups (Burke and LaFrance, 2016a,b). The need for further study into determining factors for stereotype content in relation to bisexual groups is not limited to perceiver sexual orientation, but also includes salience of the assumed gender of sexual partners (Zivony and Saguy, 2018) and the impact of essentialist views on sexuality (Hubbard and de Visser, 2015). This can provide further information on how gender is given meaning by a perceiver depending on the interplay of gender, sexual orientation, gender of partner, and perceiver attitudes.

A further avenue of inquiry is to investigate how stereotypes at the intersection of gender and sexual orientation relate to other suggested dimensions of stereotype content. A competing model to the SCM is the Agency, Beliefs, Communion (ABC) model of stereotypes. The ABC model suggests that the key dimensions used in group evaluation are agency/socioeconomic success and conservative-progressive beliefs, while communion is an emergent property of a group’s perceived agency (Koch et al., 2016). Available research on sexual minority groups using all three of these dimensions indicate that lesbian women, gay men, and “bisexuals” are all stereotyped as low in agency and high on progressive beliefs. Ratings of communion differ between studies samples, with “bisexuals” receiving low ratings, gay men receiving high ratings, and lesbian women shifting between the two (Koch et al., 2016). Available studies on the ABC model utilize the same paradigm as research within the SCM paradigm, in which only groups identified as particularly culturally salient are included (see e.g., Koch et al., 2016, 2020). There is therefore a distinct lack of dedicated research on how the additional dimension of conservative-progressive beliefs relate to stereotype content at the intersection of gender and sexual orientation that future research should rectify.

Due to the changing nature of stereotype content in response to societal developments (see for instance, Diekman and Eagly, 2000; Hentschel et al., 2019; Sendén et al., 2019; Eagly et al., 2020), there is a need to conduct ongoing studies into stereotype content. The current study, therefore, provides insight into the current conceptualization of the dynamic relation between gendered attributes and sexual orientations in Sweden: a snapshot rather than a definitive image. Furthermore, the use of multiple forms of measurement in studies on stereotype content can provide greater insight into the limits of the concept under study and sampling others than student populations can give a more general view of stereotype content. Future studies on implicit associations would benefit from using several types of implicit tests to investigate if the lack of differential implicit associations in the current study is a methodological artifact or due to the complexity of associations.

A final avenue for future research is to investigate further what the implications are of intersectional invisibility and hypervisibility in terms of stereotype content. Intersectional invisibility has been linked to several negative consequences (e.g., lack of recognition or resources), but it could also potentially have beneficial effects in certain contexts (e.g., being less of a target of direct discrimination; Purdie-Vaughns and Eibach, 2008). Similarly, the hypervisibility of being a prototypical member of a marginalized group, for instance, a gay man, brings with it negative consequences (e.g., higher risk of legal discrimination), but also some benefits (e.g., higher likelihood to reach a leader position within the marginalized group; Purdie-Vaughns and Eibach, 2008). Research on recruitment for leadership positions has found either that lesbian women face a higher degree of discrimination in recruitment than gay men do (Fasoli and Hegarty, 2019), that lesbian women and gay men face equal amounts of discrimination (Fasoli et al., 2017), or that lesbian women and gay men are as equally likely to be hired as their heterosexual counterparts (Niedlich and Steffens, 2015). Moreover, gay men who display gender non-conformity have been evaluated less positively as leaders than gender conforming gay men (de Cristofaro et al., 2020; Salvati et al., 2021). We have found no similar research on the effect on leadership potential of gender conformity of lesbian women. However, both lesbian women and gay men have been rated as respectively higher in task competence and social skills in a recruitment situation than heterosexual women and men, but this high competence in gender non-stereotypical skills did not lead to higher hireability judgments (Niedlich and Steffens, 2015). Beyond leadership, correspondence studies on recruitment discrimination show that lesbian women and gay men, compared to heterosexual women and men, have generally lower chances of receiving an interview invitation following a job application, and this bias has a gendered dimension. Lesbian women face particular discrimination in professions with majority women employees, while gay men face particular discrimination in professions with majority men employees (Ahmed et al., 2013; Drydakis, 2015). Furthermore, sexual minority individuals who exhibit higher degrees of gender non-conformity are more likely to have experienced prejudiced events throughout their lifetime (Thoma et al., 2021), and are more likely to be met with more negative attitudes from both heterosexual (Cohen et al., 2009) and homosexual individuals (Salvati et al., 2018). From this research, it is clear that the perceived gender inversion of sexual minority groups has real and negative consequences for the lives of sexual minority individuals, and that sexual minority individuals who are seen as gender non-conforming are particularly subjected to societal discrimination. There is thus a clear need for further research on the implications of differing or overlapping stereotype content in relation to general social groups.

### Concluding Remarks

Intersectional invisibility (Purdie-Vaughns and Eibach, 2008) of individuals with more than one subordinate group identity has previously been found in relation to the intersection of gender and ethnicity (Ghavami and Peplau, 2013). The current study extended these findings to the intersection of gender and sexual orientation by showing that stereotype content for “women” and “men” only overlaps with that of heterosexual women and men, but not with that of subgroups that do not conform to a heteronormative ideology. This relation between general gender categories and heterosexual categories invites us to consider how gender and sexual orientation rely upon each other. Consider the term “gender inversion” used throughout the literature (and this article) to discuss the stereotype content of sexual minorities. What does this term imply about the relation that these groups have to gender as a binary structure, are lesbian and bisexual women not women and are gay and bisexual men not men? Speaking directly to this point, Wittig (1992a, p. 13) stated that “The refusal to become (or to remain) heterosexual always meant to refuse to become a man or a woman, consciously or not.” The results of the current study support the notion that without heterosexuality, gender loses much of its established meaning. In the case of bisexual groups, the lack of a clear gender of attraction further seems to rip gender from its moorings, denying the guidance that is present for homosexual groups in how to “invert” gendered expectations. Investigating how gender stereotype content relates to sexual orientation subgroups thus identifies the organizing role that heterosexuality plays in structuring gender as expressed in the current binary gender system.

## Data Availability Statement

The raw data supporting the conclusions of this article will be made available by the authors, without undue reservation.

## Ethics Statement

Ethical review and approval was not required for the study on human participants in accordance with the local legislation and institutional requirements. The patients/participants provided their written informed consent to participate in this study.

## Author Contributions

AK conceptualized the idea, collected the data, performed the statistical analyses, and wrote the manuscript. AL and FB contributed actively in the designing and planning of the studies, interpretation and discussion of results, and in revising the manuscript. All authors contributed to the article and approved the submitted version.

### Conflict of Interest

The authors declare that the research was conducted in the absence of any commercial or financial relationships that could be construed as a potential conflict of interest.

## Footnotes

^1^A note on language use is appropriate here. The current recommendations from the APA are to avoid the term “homosexual” women and men in favor of the labels lesbian women and gay men (American Psychological Association, 2020, p.153). However, because we partly study how sexual orientation categories are shared across gender groups, we have chosen to use the terms heterosexual, homosexual, and bisexual women and men when emphasizing the dynamic pattern of shared and non-shared group memberships. When referring to the cultural groups of lesbian women and gay men, we have chosen to use these recommended terms. The noun form (e.g., “bisexuals”) is only used when referring to the term used in data collection in referenced studies.

^2^www.qualtrics.com

^3^www.enkatfabriken.se
