# Supplementary material for: Stereotype Content at the Intersection of Gender and Sexual Orientation
Source: Front Psychol. 2021 Jul 15;12:713839. doi: 10.3389/fpsyg.2021.713839 (PMC8319495; doi:10.3389/fpsyg.2021.713839)
Supplement: Supplementary file 1 [file Table_1.PDF]

Stereotype Content at the Intersection of Gender and Sexual Orientation, Supplementary Materials

Supplementary materials include a breakdown of sample size by condition (Table S1), details on traits used in ratings scales (Table S2), stimuli used in Single Category Implicit Association Tests (Table S3 and S4). The supplementary materials also include results for paired *t*-tests for communion and agency within group (Table S5), exact reliability coefficients for each Single Category Implicit Association Test (Table S6) as well as means and standard deviations for reaction times in the Single Category Implicit Association Tests by target group and attribute dimension (Table S7).

Table S1. Sample size per target group

Study condition	Sample size	
	Sample 1	Sample 2
Women	94	56
Men	109	53
Heterosexual women	89	53
Heterosexual men	100	53
Homosexual women	111	55
Homosexual men	121	45
Bisexual women	104	57
Bisexual men	96	51

Table S2. Scale items used to measure attributed communality and agency

Communality	Agency
Caring$_a$ (Omhändertagande)	Self-confident$_c$ (Självsäker)
Warm$_c$ (Varm)	Stands up well under pressure$_a$ (Stresstålig)
Empathetic$_a$ (Empatisk)	Never give up easily$_a$ (Ger inte upp lätt)
Affectionate$_a$ (Tillgiven)	Have leadership qualities$_a$ (Har ledaregenskaper)
Friendly$_a$ (Vänlig)	Feel very superior$_a$ (Känner sig överlägsen)
Tolerant$_b$ (Tolerant)	Independent$_b$ (Självständig)
Good natured$_b$ (Godhjärtad)	Competitive$_b$ (Tävlingsinriktad)
Sincere$_b$ (Uppriktig)	
Just$_a$ (Rättfärdig)	Efficient$_a$ (Effektiv)
Fair$_a$ (Rättvis)	Capable$_a$ (Kapabel)
Considerate$_a$ (Hänsynsfull)	Competent$_c$ (Kompetent)
Trustworthy$_a$ (Pålitlig)	Intelligent$_c$ (Intelligent)
Reliable$_a$ (Tillförlitlig)	Clever$_a$ (Smart)

Note. Items were rated on a scale from 1 to 5 with high values representing a presence of the trait in the rated group. Suffix mark measure of origin: $_a$ = Item taken from agency/communion measures, $_b$ = item taken from warmth competence measures, $_c$ = item present in both types of measures. Swedish translation within brackets.

Table S3. Stimuli words used as associative terms for warmth, cold, competence, and incompetence the two Single Category Implicit Association Tests

Target category stimuli words			
Warmth	Cold	Competence	Incompetence
Empathetic (Empatisk)	Cold (Kylig)	Competent (Kompetent)	Useless (Oduglig)
Warm (Varm)	Cynical (Cynisk)	Efficient (Effektiv)	Lazy (Lat)
Good natured (Godhjärtad)	Heartless (Hjärtlös)	Clever (Smart)	Dumb (Dum)
Considerate (Hänsynsfull)	Ruthless (Hänsynslös)	Self-confident (Självsäker)	Stupid (Korkad)
Fair (Rättvis)	Intolerant (Intolerant)	Competitive (Tävlingsinriktad)	Slow witted (Trög)

Note. Swedish translation within brackets.

Table S4. Stimuli words used as synonyms for the study's target groups in the two Single Category Implicit Association Tests

Target category stimuli words	
**Women**	Woman (Kvinna)
	Girl (Tjej)
	Lady (Dam)
**Heterosexual women**	Heterosexual woman (Heterosexuell kvinna)
	Female heterosexual (Kvinnlig heterosexuell)
	Hetero woman (Hetero-kvinna)
**Homosexual women**	Homosexual woman (Homosexuell kvinna)
	Female homosexual (Kvinnlig homosexuell)
	Dyke (Flata)
**Bisexual women**	Bisexual woman (Bisexuell kvinna)
	Female bisexual (Kvinnlig bisexuell)
**Men**	Man (Man)
	Guy (Kille)
	Gent (Herre)
**Heterosexual men**	Heterosexual man (Heterosexuell man)
	Male heterosexual (Manlig heterosexuell)
	Hetero man (Hetero-man)
**Homosexual men**	Homosexual man (Homosexuell man)
	Male homosexual (Manlig homosexuell)
	Gay man (Bög)
**Bisexual men**	Bisexual man (Bisexuell man)
	Male bisexual (Manlig bisexuell)

Note. Swedish translation within brackets. Due to a lack of commonly used synonyms for the bisexual groups they are only represented by two synonyms.

Table S5

Results for paired t-tests within groups for communion and agency in Study 1 and 2

	t	*df*	d_{av}
**Study 1**			
Women	13.19***	93	1.74
Heterosexual women	8.53***	88	1.26
Homosexual women	-8.52***	110	0.88
Bisexual women	-4.27***	103	0.38
Men	-17.18***	108	1.85
Heterosexual men	-10.25***	99	1.31
Homosexual men	9.99***	120	1.12
Bisexual men	-5.76***	95	0.66
**Study 2**			
Women	7.35***	55	1.00
Heterosexual women	4.06***	53	0.56
Homosexual women	-0.74	53	0.08
Bisexual women	0.36	56	0.04
Men	-6.85***	52	0.98
Heterosexual men	-3.97***	52	0.57
Homosexual men	2.98**	44	0.50
Bisexual men	3.29**	50	0.39

Note. *$p < 0.05$, **$p < 0.01$, ***$p < 0.001$

Table S6. Excluded trials and participants from SC-IATs

	SC-IAT			
Reason for exclusion	Warmth-cold		Competence-incompetence	
	Full sample	Range/condition	Full sample	Range/condition
> 10% of responses < 300 ms (participants)	11	1-3	48	1-15
> 10 000 ms (trials)	47	1-9	22	0-6
> 20% error rate (participants)	21	1-5	12	1-3
M RT 2-3 SD > group mean (participants)	32	4-5	32	0-6

Note. Exclusion criteria are reported in order of application.

Table S7. Means and standard deviations for reaction times in Single Category Implicit Association Tests by condition and dimension paired with target category

	Warm		Cold		Competence		Incompetence	
	M	*SD*	*M*	*SD*	*M*	*SD*	*M*	*SD*
Women	966.79	182.34	1070.24	222.21	920.57	173.11	986.05	170.85
Men	981.09	179.54	979.64	156.51	904.32	157.44	938.62	150.2
Heterosexual women	979.09	160.57	1056.45	190.28	907.21	155.94	1015.73	181.19
Heterosexual men	954.05	138.00	1040.99	179.98	929.9	175.39	1005.19	223.68
Homosexual women	999.63	171.15	1049.31	152.54	932.5	151.1	1021.5	215.66
Homosexual men	992.61	164.16	1010.64	154.34	936.27	168.75	949.42	148.99
Bisexual women	1025.99	197.62	1082.50	206.19	916.51	193.23	1001.93	175.42
Bisexual men	920.49	170.93	999.09	220.33	913.31	186.97	904.22	157.55

Note: All reaction times are reported in milliseconds.
